# Excess risk of major vascular diseases associated with airflow obstruction: a 9-year prospective study of 0.5 million Chinese adults

**DOI:** 10.2147/COPD.S153641

**Published:** 2018-03-08

**Authors:** Om P Kurmi, Liming Li, Kourtney J Davis, Jenny Wang, Derrick A Bennett, Ka Hung Chan, Ling Yang, Yiping Chen, Yu Guo, Zheng Bian, Junshi Chen, Liuping Wei, Donghui Jin, Rory Collins, Richard Peto, Zhengming Chen

**Affiliations:** 1 Clinical Trial Service and Epidemiological Studies Unit, Nuffield Department of Population Health, University of Oxford, Oxford, UK; 2 Department of Epidemiology, School of Public Health, Peking University Health Science Center, Beijing, China; 3 Real World Evidence and Epidemiology, GlaxoSmithKline, Collegeville, PA, USA; 4 Chinese Academy of Medical Sciences, Beijing China; 5 China National Center for Food Safety Risk Assessment, Beijing, China; 6 NCDs Prevention and Control Department, Liuzhou CDC, Liuzhou, China; 7 NCDs Prevention and Control Department, Hunan CDC, Changsha, China

**Keywords:** chronic obstructive pulmonary disease, exacerbation, vascular diseases, cohort, China

## Abstract

**Background:**

China has high COPD rates, even among never-regular smokers. Little is known about nonrespiratory disease risks, especially vascular morbidity and mortality after developing airflow obstruction (AFO) in Chinese adults.

**Objective:**

We aimed to investigate the prospective association of prevalent AFO with major vascular morbidity and mortality.

**Materials and methods:**

In 2004–2008, a nationwide prospective cohort study recruited 512,891 men and women aged 30–79 years from 10 diverse localities across China, tracking cause-specific mortality and coded episodes of hospitalization for 9 years. Cox regression yielded adjusted HRs for vascular diseases comparing individuals with spirometry-defined prevalent AFO at baseline to those without.

**Results:**

Of 489,382 participants with no vascular disease at baseline, 6.8% had AFO, with prevalence rising steeply with age. Individuals with prevalent AFO had significantly increased vascular mortality (n=1,429, adjusted HR 1.29, 95% CI 1.21–1.36). There were also increased risks of hemorrhagic stroke (n=823, HR 1.18, 95% CI 1.09–1.27), major coronary events (n=635, HR 1.33, 95% CI 1.22–1.45), and heart failure (n=543, HR 2.19, 95% CI 1.98–2.41). For each outcome, the risk increased progressively with increasing COPD severity and persisted among never-regular smokers.

**Conclusion:**

Among adult Chinese, AFO was associated with significantly increased risks of major vascular morbidity and mortality. COPD management should be integrated with vascular disease prevention and treatment programs to improve long-term prognosis.

## Introduction

Airflow obstruction (AFO) is a hallmark of COPD. COPD is a common chronic condition with different phenotypes, and is also associated with multiple comorbidities.^1^ Exacerbations of COPD are important clinical events,^2^ often leading to rapid decline in lung function,^3^ worsening in quality of life and functional status,^4^ and increased risk of mortality.^5^ There is also evidence that COPD^6^ and its exacerbations^1^ are associated with increased vascular and nonvascular comorbidity,^7^–^11^ for reasons that are not fully understood. It is possible that the excess vascular and nonvascular comorbidity following COPD may be due to shared risk factors (eg, smoking and air pollution) or that COPD may represent an independent risk factor acting on the causal pathway for some types of vascular diseases.^1^

Most previous studies of COPD exacerbation with vascular and nonvascular diseases (eg, lung cancer, musculoskeletal disorders, depression, and diabetes) were conducted in Western populations, involving primarily patients in hospital settings. There is little information from population-based prospective studies, especially in low- and middle-income countries, such as China, where the underlying causes and management of COPD, as well as patterns of main vascular and nonvascular diseases, differ significantly from those in the West.^12^ In China, COPD is the third leading cause of morbidity and mortality, with high rates among even never-regular smokers.^13^ On the other hand, stroke rates, especially hemorrhagic stroke, are also high. Despite this, there is no known previous report in China about the association of COPD with vascular and nonvascular comorbidities from prospective studies.

We report relevant findings from a large nationwide prospective cohort (China Kadoorie Biobank [CKB]) of 0.5 million adults who were recruited in 2004–2008 and followed up for about 9 years through linkages to hospital records and mortality and disease registries. The main aim of the present study is to examine the associations of prevalent AFO with subsequent risk of vascular diseases, including subtypes. In addition, we also examined the association of prevalent AFO with a range of nonvascular diseases.

## Materials and methods

### Baseline survey

Detailed CKB design, procedures, and study participants have been described previously.^14^,^15^ Briefly, the baseline survey took place during 2004–2008 in 10 geographically diverse localities (Figure S1), chosen to include a range of disease incidence (including COPD) and risk exposure, taking into account local capacity and quality of mortality and disease-monitoring systems. In each area, temporary assessment clinics were set up within various local residential centers. All residents aged 35–74 years from 100–150 administrative units (rural villages or urban residential committees) in each area were invited to attend survey clinics. Approximately 30% responded, and a total of 512,891 participants were enrolled, including a few thousand volunteers just outside the aforementioned age range who attended the survey clinics, resulting an age range of 30–79 years. All participants provided written informed consent. International (Oxford Tropical Research Ethics Committee), national (Chinese Academy of Medical Sciences Ethical Review Committee), and local ethics (Chinese Center for Disease Control and Prevention Ethical Review Committee, and the scientific review boards in each of the 10 regional centers) approvals were obtained prior to the start of the survey.

At the study assessment clinics, trained health workers administered a laptop-based questionnaire that covered sociodemographic and lifestyle data, including education, income, smoking, alcohol drinking, self-reported physical activities, diet, and medical history (including self-reported physician diagnoses of COPD, vascular diseases, and a range of other diseases), and measured lung function, height, body weight, bioimpedance, blood pressure, and heart rate, and took a blood sample for long-term storage.^16^

### Assessment of prevalent AFO at baseline

We decided to use the general term “AFO” instead of “COPD”, as all the results are based on prebronchodilator spirometry. Forced expiratory volume in 1 second (FEV_1_) and forced vital capacity (FVC) were measured using a handheld microspirom-eter (MS01; CareFusion, San Diego, CA, USA) by respiratory technicians following recommended procedures. Participants were asked to make a few practice blows under the supervision of the respiratory technician, after which the results of two successful maneuvers (as judged by the technician) were recorded. Spirograms of a small number of participants (n=1,586) who also took part in a resurvey were reviewed as per European Respiratory Society/American Thoracic Society guidelines^17^ for quality and reproducibility. Among those reviewed, 84% had acceptable spirometry. The highest FEV_1_ and FVC values were used, not necessarily from the same blows, as recommended by ERS/ATS guidelines.^17^ Presence of AFO was defined as having measured FEV_1_/FVC < lower limit of normal (LLN) prebronchodilator lung function, based on the predicted values from the global lung function initiative (GLI) 2012 equations, and AFO grades were classified as follows: grade 1= FEV_1_/FVC < LLN (*z*-score of FEV_1_ −2 to 1), grade 2/3= FEV_1_/FVC < LLN (*z*-score of FEV_1_ −3 to −2), and grade 4/5= FEV_1_/FVC < LLN (*z*-score of FEV_1_ < −3).^18^ AFO grades were also classified based on the percentage predicted value of FEV_1_ for comparison with earlier published studies.

### Follow-up for mortality and morbidity

The vital status of each participant was monitored regularly through the China National Center for Disease Control and Prevention disease-surveillance point system, and checked annually against local residential records and health insurance records, and by active confirmation through street committee or village administrators. Cause of death from official death certificates was supplemented by a review of available medical records and coded using the ICD-10 by trained staff who were blinded to baseline information. For four major diseases (stroke, coronary heart disease, diabetes, and cancer), information on incidence was also collected through linkage with existing local disease registries. Electronic record linkage was also established with the China national health insurance system, which records the details of all hospitalized events (including description and ICD-10 code) and procedures. All records for vascular diseases from any source were checked and standardized.

For the present study, the primary vascular diseases of interest were vascular death and incident ischemic stroke (IS), hemorrhagic stroke, major coronary events (MCEs), heart failure (HF), and major vascular events. In addition, several other disease outcomes were considered, including respiratory (pneumonia, other respiratory diseases, and lung cancer), noncardiovascular, and nonrespiratory diseases (eg, chronic kidney diseases, nonlung cancer, fracture, rheumatoid arthritis, and diabetes). ICD-10 codes defining these disease outcomes are listed in Table S1.

By January 1, 2016, 37,289 (7.3%) participants had died (including 4,752 in those with prior vascular diseases) and 4,875 (1%) were lost to follow-up. The main analysis excluded 23,113 participants (10,061 men and 13,052 women) with self-reported vascular diseases at baseline. Furthermore, 396 participants (202 men and 194 women) with implausible spirometry were excluded. After these exclusions, 489,382 participants (200,157 men and 289,225 women) remained.

### Statistical analyses

Means and prevalence of baseline characteristics were calculated according to baseline AFO status defined by spirometry criteria and standardized for age in 5-year groups, region, and sex of the baseline population. Cox regression was used to estimate HRs and 95% CIs for disease risks, comparing participants with AFO versus those without, adjusting for age at risk (5-year groups), education (none/primary, secondary, tertiary), household income (CH¥; <5,000, 5,000–9,9900, 10,000–19,990, ≥20,000 per year), smoking (never-regular, ex-regular, current-regular), alcohol consumption (never-regular, ex-regular, current-regular), body mass index (kg/m^2^), and physical activity (metabolic-equivalent-of-task hours/day). For analyses involving more than two exposure categories, the floating absolute risk method was used, which provides the variance of the logarithm of the HR (ie, to compute a CI for the HR) for each category (including the reference category) to facilitate comparisons among the different exposure categories.^19^ Separate analyses were conducted for AFO grades, defined according to *z*-score for FEV_1_.^18^ Additional analyses for AFO grades categorized by percentage predicted FEV_1_ were carried out to compare our results with those from previously published studies. Sensitivity analyses were carried out to see associations in never-regular smokers and sex separately. All analyses used SAS version 9.3.

## Results

Of the 489,382 participants, 6.8% had AFO at baseline, with age-standardized prevalence higher in men than in women (7.3% versus 6.4%) and in rural than in urban regions (8.4% versus 4.9%). Compared with participants without AFO, those with AFO were older, not as well educated, and had lower income, lower body mass index, and lower physical activity. AFO was also associated with current smoking and self-reported poor health status (Table 1).

**Figure 1:**
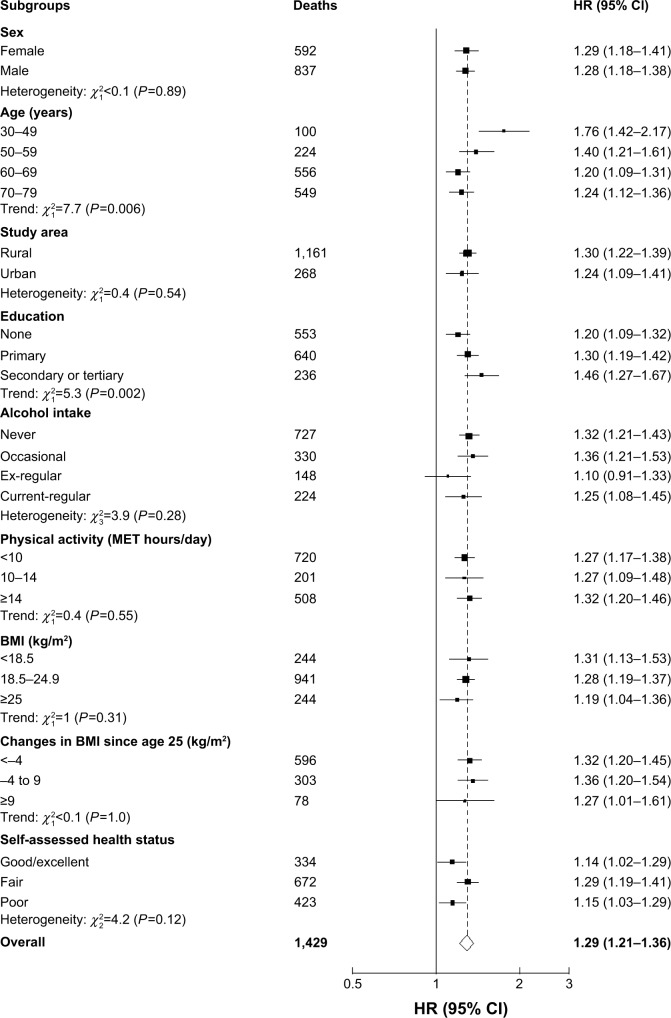
Adjusted HRs for vascular mortality by baseline AFO status. **Notes:** Stratified by sex, age, study area, education, smoking status, alcohol consumption, physical activity, BMI, and self-assessed health status. HRs and 95% CIs are floated measures. Solid squares represent the HR, with area inversely proportional to the variance of the log HR. Horizontal lines represent the corresponding 95% CIs, which are floated measures. The dotted line represents the overall HR. **Abbreviations:** AFO, airflow obstruction; BMI, body mass index; MET, metabolic equivalent of task; FEV_1_, forced expiratory volume in 1 second; FVC, forced vital capacity.

During 4.4 million person-years of follow-up (mean 9 years), 11,599 participants without self-reported vascular disease at baseline subsequently died of vascular disease. Individuals with prevalent AFO at baseline had significantly increased risk of vascular mortality, with an adjusted HR of 1.29 (95% CI 1.21–1.36) (Table 2). Similarly, there were also significantly increased risks of incident vascular diseases, including hemorrhagic stroke (HR 1.18, 95% CI 1.09–1.27), MCEs (HR 1.33, 95% CI 1.22–1.45), and HF (HR 2.19, 95% CI 1.98–2.41), but not for IS (HR 0.97, 95% CI 0.93–1.02). Aggregating all incident major vascular diseases, there was a 27% (HR 1.27, 95% CI 1.23–1.32) excess risk among participants with AFO. For vascular mortality, the excess risk appeared to be similar across different subgroups of participants (Figure 1), except among those with younger age and had higher education.

**Figure 2:**
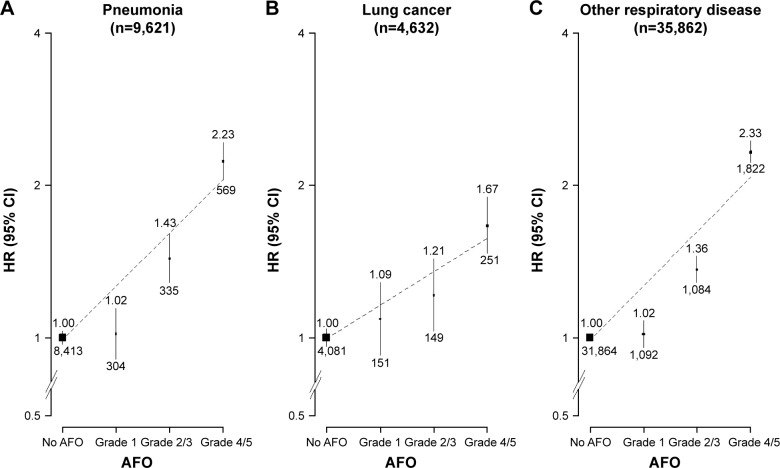
Adjusted HRs for respiratory diseases by severity of AFO. **Notes:** (**A**) Pneumonia: ICD 10 codes J12–J18. (**B**) Lung cancer: ICD 10 codes C33–C34. (**C**) Other respiratory diseases: ICD 10 codes J00–J99 excluding J12–J18 and J41–J44. Black boxes represent RRs, with area inversely proportional to variance of log RR, and vertical lines represent corresponding 95% CIs. Values above the vertical lines are HRs, and values below them are the number of events in respective categories. **Abbreviations:** AFO, airflow obstruction; ICD 10, International Classification of Diseases Version 10.

As expected, participants with AFO at baseline had significantly increased risk of major respiratory diseases during follow-up, with adjusted HRs of 1.53 (95% CI 1.44–1.63) and 1.5 (95% CI 1.45–1.55) for pneumonia and other respiratory diseases, respectively (Table 2 and Figure 2). With the exception of lung cancer (HR 1.34, 95% CI 1.22–1.47) and marginally for fracture (HR 1.06, 95% CI 1–1.13), there was no evidence of any significant excess risk associated with prevalent AFO for a range of other nonvascular and nonrespiratory diseases that were considered (eg, rheumatoid arthritis, diabetes, chronic kidney disease) (Table 2).

**Figure 3:**
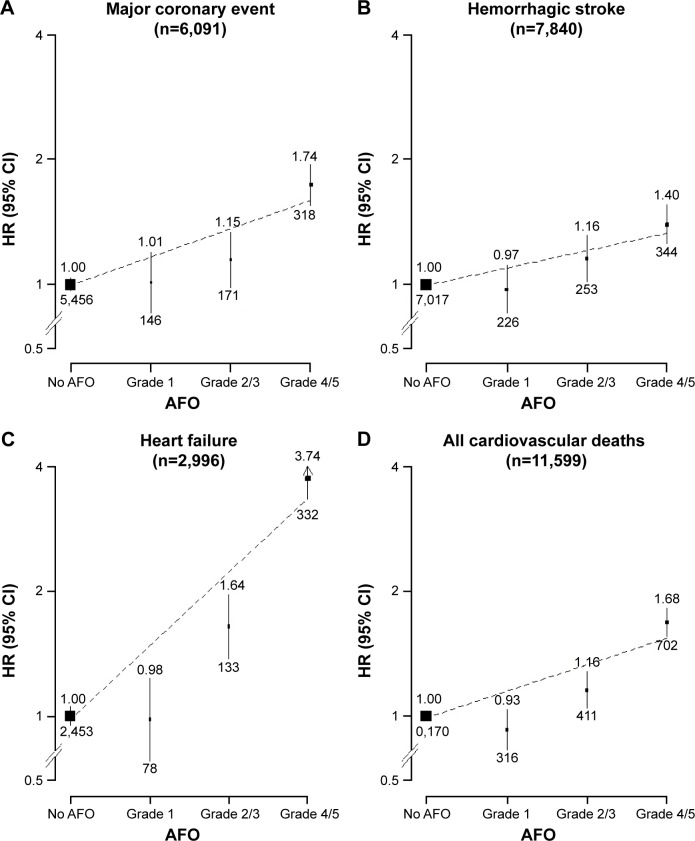
Adjusted HRs for major cardiovascular diseases by severity of AFO. **Notes:** (**A**) Major coronary event, for non-fatal: ICD 10 codes MI I21–I23; or for fatal: IHD ICD 10 codes I20–I25. (**B**) Hemorrhagic stroke, ICD 10 code I61. (**C**) Heart failure: ICD 10 codes I50. (**D**) All cardiovascular deaths: ICD 10 codes I00–I99. Black boxes represent RRs, with area inversely proportional to variance of log RR, and vertical lines represent corresponding 95% CIs. Values above the vertical lines are HRs, and values below them are the number of events in respective categories. **Abbreviations:** AFO, airflow obstruction; ICD 10, International Classification of Diseases Version 10.

The excess risks of vascular outcomes were greater among participants with more severe AFO at baseline (Tables 3 and S2). For vascular mortality, adjusted HRs were 0.93 (95% CI 0.83–1.04), 1.16 (95% CI 1.05–1.28), and 1.68 (95% CI 1.56–1.81) for AFO grades 1, 2/3, and 4/5, respectively (*P*<0.001). With the exception of IS, a similar trend was also seen for other vascular diseases, including hemorrhagic stroke, MCEs, and HF (Figure 3). No apparent trend was observed for other nonvascular and nonrespiratory conditions, with the exception of lung cancer (Table 3).

Sensitivity analyses carried out in never-regular smokers did not change the result significantly, except that the excess risk of the majority of vascular mortality was attenuated marginally in those with AFO (Table S3) at baseline and also those with higher grades (Table S4). Overall, there was no significant excess risk of lung cancer in nonsmoking participants with spirometry-defined AFO (Table S3), but the risk of lung cancer significantly increased in those with AFO grade 4/5 (Table S4). Similarly, sensitivity analyses carried out in males and females suggested only that the risk of vascular and nonvascular mortality associated with AFO was somewhat greater in males than in females (Tables S5 and S6), particularly for those with higher AFO grades (Tables S7 and S8).

## Discussion

The present study provides large-scale prospective evidence that individuals with prevalent AFO have increased risk of major vascular diseases, with risk increased proportionally with increasing severity of AFO. These effects were not accounted for by other demographic-, lifestyle-, or health-related factors, and appeared to be consistent across different subgroups of individuals and also among never-smokers. We deliberately used the more general term “AFO”, due to the lack of postbronchodilator lung function measurement.

Our study has several methodological strengths. It used a prospective study design and included a large sample from 10 diverse localities across China, thereby increasing generalizability. Disease outcomes were ascertained objectively through linkage to mortality registries and hospital records, minimizing differential misclassification due to recall or interviewer bias in assessing disease outcomes. The findings are statistically robust, as the main analyses excluded all participants with prevalent vascular diseases and also adjusted for a range of potential confounding factors, including smoking status. We used measured lung function to define prevalent AFO, which in contrast to many previous studies^12^,^20^–^22^ that only used self-reported diagnosis by physician should provide better assessment of the actual prevalence at the community level. Adults with early stages of AFO are less likely to seek care from a doctor, and hence would miss the opportunity to receive a diagnosis, whereas our study protocol with a systematic screening spirometry test was likely to capture cases with early signs of lower lung function. Nevertheless, given the lack of postbronchodilator spirometry data to differentiate asthma cases, it is possible our definition of AFO may have included some false-positive cases. However, it is known that lung function in at least 10%–15% of COPD cases is likely to be partially reversible postbronchodilator and that the prevalence of adult asthma is very low (<1%) in China,^23^–^25^ suggesting that the potential misclassification is likely to be small. The sensitivity analysis restricted to never-regular smokers did not change the results significantly, suggesting little residual confounding from active smoking.

Several previous studies of mostly Western populations have investigated the associations of COPD with a range of vascular and nonvascular diseases.^9^,^21^,^22^,^26^,^27^ Most of these studies were small, used retrospective study designs, and included mainly hospital patients with severe COPD; some also lacked appropriate control for potential confounding factors (eg, smoking and socioeconomic status).^21^,^28^,^29^ Moreover, there were also large differences in the criteria used for defining COPD and major vascular outcomes. Consequently, the previous study findings have been inconsistent both qualitatively and quantitatively, with a few studies reporting no significant association of COPD with certain cardiovascular diseases, such as stroke,^9^,^30^ whereas others^20^,^31^ reported more than threefold increased risk. Although our study was consistent with several earlier studies in showing positive associations between COPD and risk of vascular diseases,^12^,^20^,^27^,^28^,^32^,^33^ the strength of the associations observed in our study appeared to be more modest and increased marginally for some outcomes, such as vascular mortality, when restricted to never-regular smokers. However, when the analyses were restricted to those with more severe AFO, our risk estimates were generally compatible with those other studies, which included only participants recruited directly from hospital settings with more severe COPD.^20^,^28^

Our study shows that with the exception of IS, the risk of major vascular diseases increased significantly among those with AFO grade 2 or higher. In a joint analysis of two prospective cohorts in the US involving 20,296 people with 12,877 COPD,^34^ individuals with GOLD stages 2 and 3/4 COPD had 2.2- and 2.4-fold risk of cardiovascular disease, respectively, compared with those without COPD. For HF, there was a two- to sixfold increased risk following hospitalization for an acute exacerbation of COPD, similar to that observed in the present study. The mechanism underlying the association with HF is unclear, but one possibility could be that serious hypoxia associated with COPD causes excess strain on the right ventricle over time, leading to dilation and stretching and ultimately resulting in HF.^35^

In our study, the observed association of AFO with stroke appeared to vary by stroke subtype, with significant excess risk for hemorrhagic stroke, but not IS. A large retrospective cohort study of 1.2 million individuals selected from primary care records in the UK^28^ reported over threefold increased risk for stroke. Although no information was available on stroke subtypes in that study, most would probably have involved ischemic rather than hemorrhagic stroke, given 85% of all strokes in the UK are ischemic and only 15% hemorrhagic.^36^

The association between AFO and vascular diseases appeared to be at least as strong in never-regular smokers as ever-regular smokers, suggesting other proinflammatory agents, such as air pollution and occupational exposures, could have played a role.^37^,^38^ Although proportional increases in vascular mortality and morbidity were greater in younger adults, absolute risks were higher in the elderly, due to higher absolute disease rates. Similar age-related findings have also been reported in other studies in the UK^28^ and Denmark.^33^

In our analyses, we also included some respiratory conditions (ie, pneumonia and lung cancer) and nonrespiratory and nonvascular diseases (eg, cancers other than lung, fracture, rheumatoid arthritis, diabetes, and chronic kidney disease) to validate indirectly our findings on vascular diseases as reported in earlier literature. Compared to those without AFO, individuals with prevalent AFO had significant excess risk of other respiratory diseases, but little excess risk of nonrespiratory conditions. The excess risk of lung cancer was not significant when restricting the analysis to never-regular smokers, suggesting that the association may be due to confounding by smoking and that AFO is not an independent risk factor for lung cancer, particularly in those with lower AFO grades (≤3). Previous studies have also reported a positive association of COPD prevalence with diabetes^28^,^33^ and rheumatoid arthritis.^28^ Possible explanations for these associations include shared risk factors, such as sedentary lifestyles and systematic inflammation.^7^ In a joint analysis of 20,296 people from two prospective cohort studies in the US,^34^ individuals with COPD had up to 1.5-fold the risk of diabetes compared to those without COPD. Similarly, another meta-analysis of 16 randomized controlled trials with 17,513 COPD patients reported 27% higher risk of fractures in those with long-term use of inhaled corticosteroids, similar to that in another pooled analyses of seven observational studies of 69,000 participants.^39^ In our study, detailed information on medication was not prospectively collected, so we were unable to test this hypothesis directly.

In summary, our prospective cohort study found that AFO was strongly associated with cardiovascular risk in both smoking and nonsmoking Chinese adults. Our findings suggest a shift toward holistic approaches to the diagnosis, treatment plans, and management of commonly co-occurring conditions, rather than focusing on single diseases, for optimal improvement in health outcomes. Clinical trials in COPD patients demonstrating the impact of COPD treatments on vascular disease outcomes are needed to understand further the nature of the association and inform clinical decision-making. Targeting common risk and prognostic factors for COPD and vascular diseases more aggressively (eg, smoking, environmental exposures, physical activity) is sensible to reduce and prevent premature morbidity, associated disability, and mortality.

## Researchers

Institutions and members thereof involved in this study are listed in Table 4.

## Figures and Tables

**Table 1:** Baseline characteristics of participants by baseline AFO status (defined by spirometry criteria only)

Characteristics	AFO at baseline (%)		
	No (n=456,143)	Yes (n=33,239)	Total (n=489,382)
**Sex**			
Female	59.3	55.7	59.1
Male	40.7	44.3	40.9
**Region**			
Urban	45.0	32.3	44.0
Rural	55.0	67.7	56.0
**Age at baseline (years)**			
30–49	47.4	36.7	46.6
50–59	30.9	28.5	30.8
60–69	16.3	23.2	16.8
70–79	5.5	11.5	5.8
Mean ± SD	50.9±10.5	54±11.4	51.1±10.5
**Highest education completed**			
No formal	20.1	23	20.3
Primary	31.6	32.1	31.6
Secondary or tertiary	48.3	44.9	48.1
**Annual household income (CH¥)**			
<5,000	9.4	12.6	9.6
5,000–9,999	18.1	20.1	18.2
10,000–19,999	29.1	30.2	29.1
≥20,000	43.4	37.2	43.1
**BMI (kg/m^2^)**			
<18.5	4.0	7.9	4.4
18.5–25	62.3	67.4	62.6
≥25	33.6	24.8	33
**Smoking category**			
Never-regular	67.5	64.2	67.2
Ex-regular	5.7	6.7	5.8
Current-regular	26.8	29.1	27
**Alcohol intake**			
Never	45.6	48.4	45.8
Occasional	35.1	32.1	34.9
Ex-regular	3.8	4.5	3.9
Regular	15.5	15	15.4
**Physical activity (MET hours/day)**			
<10	22.9	24.6	23
10–14	14.3	14.4	14.3
≥14	62.8	61.0	62.7
**Lung function (mean ± SD)**			
FEV_1_/FVC (%)	85.8±6.4	65.4±7.8	84.4±8.4
FEV_1_ (L)	2.3±0.6	1.7±0.7	2.2±0.7
FVC (L)	2.7±0.7	2.6±0.9	2.7±0.8
Self-reported diseases at baseline*			
Hypertension	10.6	9.4	10.5
Diabetes	2.9	2.5	2.9
Rheumatic heart disease	0.2	0.3	0.2
Asthma	0.4	3.1	0.5
Tuberculosis	1.4	2.7	1.5
Cancer	0.5	0.6	0.5
**Self-assessed health status**			
Good/excellent	47.0	40.2	46.5
Fair	43.9	45.6	44
Poor	9.2	14.3	9.6

**Notes:** All participants reporting prior doctor-diagnosed coronary heart disease or stroke at baseline excluded; all data standardized to age, sex, and study area as appropriate;

* All *P*-values for differences between participants with and without baseline AFO are <0.001 with the exception of self-reported diseases at baseline.

**Abbreviations:** AFO, airflow obstruction; BMI, body mass index; MET, metabolic equivalent of task; FEV_1_, forced expiratory volume in 1 second; FVC, forced vital capacity.

**Table 2:** Events, adjusted event rates, and HRs for selected disease outcomes by prevalent AFO*

	No prevalent AFO		Prevalent AFO		HR (95% CI)‡
	Events	Rate/1,000 person-years#	Events	Rate/1,000 person-years#	
**Respiratory**					
Pneumonia	8,413	2.15	1,208	3.86	1.53 (1.44–1.63)
Other	31,864	8.19	3,998	14.17	1.50 (1.45–1.55)
**Cardiovascular**					
Ischemic stroke	29,928	7.67	2,018	7.39	0.97 (0.93–1.02)
Hemorrhagic stroke	7,017	1.80	823	2.17	1.18 (1.09–1.27)
Major coronary event	5,456	1.42	635	1.83	1.33 (1.22–1.45)
Heart failure	2,453	0.64	543	1.33	2.19 (1.98–2.41)
All cardiovascular deaths	10,170	2.71	1,429	3.69	1.29 (1.21–1.36)
Major vascular events	43,262	11.3	4,458	13.51	1.27 (1.23–1.32)
**Others**					
Lung cancer	4,081	1.03	551	1.59	1.34 (1.22–1.47)
Nonlung cancer	17,727	4.44	1,614	4.60	1.01 (0.95–1.06)
Fracture	11,568	2.90	1,107	3.00	1.06 (1–1.13)
Rheumatoid arthritis	1,052	0.25	83	0.29	1.09 (0.86–1.37)
Diabetes	12,905	3.24	1,066	3.04	0.96 (0.9–1.02)
Chronic kidney disease	1,594	0.39	118	0.37	1.04 (0.86–1.26)

**Notes:**

* AFO defined as FEV_1_/FVC < lower limit of normal, based on baseline lung function measurement;

# rates adjusted for age, sex, and region;

‡ adjusted for age, sex, region, smoking, education, BMI, physical activity, and alcohol intake and stratified by age at risk, sex, and study area.

**Abbreviations:** AFO, airflow obstruction; BMI, body mass index; FEV_1_, forced expiratory volume in 1 second; FVC, forced vital capacity.

**Table 3:** Event, adjusted event rates, and HRs for selected disease outcomes by different grades of prevalent AFO*

Incident cases	No AFO			AFO – grade 1			AFO – grade 2/3			AFO – grade 4/5		
	Events	Rate#	HR (95% CI)‡	Events	Rate#	HR (95% CI)‡	Events	Rate#	HR (95% CI)‡	Events	Rate#	HR (95% CI)‡
**Respiratory**												
Pneumonia	8,413	2.15	1 (0.97–1.03)	304	2.10	1.02 (0.91–1.14)	335	3.44	1.43 (1.29–1.60)	569	6.22	2.23 (2.05–2.43)
Other respiratory	31,864	8.19	1 (0.99–1.01)	1,092	8.31	1.02 (0.96–1.08)	1,084	12.84	1.36 (1.28–1.45)	1,822	25.08	2.33 (2.22–2.44)
**Cardiovascular**												
Ischemic stroke	29,928	7.67	1 (0.98–1.02)	631	7.41	0.91 (0.84–0.99)	647	7.47	1.06 (0.98–1.15)	740	7.09	0.95 (0.88–1.02)
Hemorrhagic stroke	7,017	1.80	1 (0.97–1.03)	226	1.55	0.97 (0.85–1.11)	253	2.13	1.16 (1.02–1.31)	344	2.56	1.40 (1.26–1.55)
Major coronary event	5,456	1.42	1 (0.97–1.03)	146	1.26	1.01 (0.86–1.19)	171	1.46	1.15 (0.99–1.33)	318	2.46	1.74 (1.55–1.94)
Heart failure	2,453	0.64	1 (0.95–1.05)	78	0.56	0.98 (0.79–1.23)	133	0.85	1.64 (1.38–1.95)	332	2.46	3.74 (3.35–4.17)
All cardiovascular deaths	10,170	2.71	1 (0.98–1.03)	316	2.40	0.93 (0.83–1.04)	411	3.06	1.16 (1.05–1.28)	702	5.24	1.68 (1.56–1.81)
Major vascular event	43,262	11.3	1 (0.99–1.01)	1,046	10.61	0.9 (0.85–0.96)	1,210	11.87	1.14 (1.08–1.21)	2,202	17.38	1.71 (1.64–1.79)
**Other**												
Lung cancer	4,081	1.03	1 (0.96–1.04)	151	1.20	1.09 (0.93–1.28)	149	1.43	1.21 (1.03–1.43)	251	2.16	1.67 (1.47–1.89)
Nonlung cancer	17,727	4.44	1 (0.98–1.02)	595	4.45	1.02 (0.94–1.10)	471	4.97	1.02 (0.93–1.11)	548	4.56	0.99 (0.91–1.07)
Fracture	11,568	2.90	1 (0.98–1.02)	439	3.06	1.06 (0.96–1.16)	321	2.80	1.03 (0.92–1.15)	346	3.01	1.09 (0.98–1.22)
Rheumatoid arthritis	1,052	0.25	1 (0.92–1.08)	31	0.26	1.02 (0.72–1.45)	24	0.27	1.06 (0.71–1.59)	28	0.36	1.20 (0.83–1.74)
Diabetes	12,905	3.24	1 (0.98–1.02)	398	2.52	0.84 (0.76–0.93)	313	3.15	0.94 (0.84–1.05)	355	3.68	1.16 (1.04–1.29)
Chronic kidney disease	1,594	0.39	1 (0.94–1.07)	52	0.35	1.25 (0.95–1.65)	31	0.37	0.91 (0.64–1.29)	35	0.31	0.92 (0.66–1.28)

**Notes:**

* AFO defined as FEV_1_/FVC < LLN based on baseline lung function measurement; grade 1= FEV_1_/FVC < LLN (*z*-score of FEV_1_ −2 to 1); grade 2/3= FEV_1_/FVC < LLN (*z*-score of FEV_1_ −3 to −2); grade 4/5= FEV_1_/FVC < LLN (*z*-score of FEV_1_ < −3);

# rates (per 1,000 person-years) adjusted for age, sex, and study area;

‡ HRs adjusted for smoking, education, body mass index, physical activity level, and alcohol intake and stratified by age at risk, sex, and study area.

**Abbreviations:** AFO, airflow obstruction; FEV_1_, forced expiratory volume in 1 second; FVC, forced vital capacity; LLN, lower limit of normal.

**Table 4:** Members of the China Kadoorie Biobank collaborative group

International Steering Committee	Junshi Chen, Zhengming Chen (PI), Robert Clarke, Rory Collins, Yu Guo, Liming Li (PI), Jun Lv, Richard Peto, Robin Walters
International Coordinating Center,Oxford	Daniel Avery, Ruth Boxall, Derrick Bennett, Yumei Chang, Yiping Chen, Zhengming Chen, Robert Clarke, Huaidong Du, Simon Gilbert, Alex Hacker, Michael Holmes, Christiana Kartsonaki, Rene Kerosi, Om Kurmi, Garry Lancaster, Kuang Lin, John McDonnell, Iona Millwood, Qunhua Nie, Jayakrishnan Radhakrishnan, Paul Ryder, Sam Sansome, Dan Schmidt, Rajani Sohoni, Becky Stevens, Iain Turnbull, Robin Walters, Jenny Wang, Lin Wang, Neil Wright, Ling Yang, Xiaoming Yang
National Coordinating Center, Beijing	Zheng Bian, Yu Guo, Xiao Han, Can Hou, Jun Lv, Pei Pei, Chao Liu, Biao Jing, Yunlong Tan, Canqing Yu
**Regional coordinating centers**	
Qingdao CDC	Zengchang Pang, Ruqin Gao, Shanpeng Li, Shaojie Wang, Yongmei Liu, Ranran Du, Yajing Zang, Liang Cheng, Xiaocao Tian, Hua Zhang, Yaoming Zhai, Feng Ning, Xiaohui Sun, Feifei Li
Licang CDC	Silu Lv, Junzheng Wang, Wei Hou
Heilongjiang Provincial CDC	Mingyuan Zeng, Ge Jiang, Xue Zhou
Nangang CDC	Liqiu Yang, Hui He, Bo Yu, Yanjie Li, Qinai Xu, Quan Kang, Ziyan Guo
Hainan Provincial CDC	Dan Wang, Ximin Hu, Hongmei Wang, Jinyan Chen, Yan Fu, Zhenwang Fu, Xiaohuan Wang
Meilan CDC	Min Weng, Zhendong Guo, Shukuan Wu, Yilei Li, Huimei Li, Zhifang Fu
Jiangsu Provincial CDC	Ming Wu, Yonglin Zhou, Jinyi Zhou, Ran Tao, Jie Yang, Jian Su
Suzhou CDC	Fang Liu, Jun Zhang, Yihe Hu, Yan Lu, Liangcai Ma, Aiyu Tang, Shuo Zhang, Jianrong Jin, Jingchao Liu
Guangxi Provincial CDC	Zhenzhu Tang, Naying Chen, Ying Huang
Liuzhou CDC	Mingqiang Li, Jinhuai Meng, Rong Pan, Qilian Jiang, Jian Lan, Yun Liu, Liuping Wei, Liyuan Zhou, Ningyu Chen, Ping Wang, Fanwen Meng, Yulu Qin, Sisi Wang
Sichuan Provincial CDC	Xianping Wu, Ningmei Zhang, Xiaofang Chen, Weiwei Zhou
Pengzhou CDC	Guojin Luo, Jianguo Li, Xiaofang Chen, Xunfu Zhong, Jiaqiu Liu, Qiang Sun
Gansu Provincial CDC	Pengfei Ge, Xiaolan Ren, Caixia Dong
Maiji CDC	Hui Zhang, Enke Mao, Xiaoping Wang, Tao Wang, Xi Zhang
Henan Provincial CDC	Ding Zhang, Gang Zhou, Shixian Feng, Liang Chang, Lei Fan
Huixian CDC	Yulian Gao, Tianyou He, Huarong Sun, Pan He, Chen Hu, Xukui Zhang, Huifang Wu
Zhejiang Provincial CDC	Min Yu, Ruying Hu, Hao Wang
Tongxiang CDC	Yijian Qian, Chunmei Wang, Kaixu Xie, Lingli Chen, Yidan Zhang, Dongxia Pan, Qijun Gu
Hunan Provincial CDC	Yuelong Huang, Biyun Chen, Li Yin, Huilin Liu, Zhongxi Fu, Qiaohua Xu
Liuyang CDC	Xin Xu, Hao Zhang, Huajun Long, Xianzhi Li, Libo Zhang, Zhe Qiu
